# Decoding *Cryptococcus*: From African biodiversity to worldwide prevalence

**DOI:** 10.1371/journal.ppat.1012876

**Published:** 2025-02-03

**Authors:** Marco A. Coelho, Márcia David-Palma, Janneke Aylward, Nam Q. Pham, Cobus M. Visagie, Taygen Fuchs, Neriman Yilmaz, Francois Roets, Sheng Sun, John W. Taylor, Brenda D. Wingfield, Matthew C. Fisher, Michael J. Wingfield, Joseph Heitman

**Affiliations:** 1 Department of Molecular Genetics and Microbiology, Duke University Medical Center, Durham, North Carolina, United States of America; 2 Department of Biochemistry, Genetics and Microbiology, Forestry and Agricultural Biotechnology Institute (FABI), University of Pretoria, Pretoria, South Africa; 3 Department of Conservation Ecology and Entomology, Stellenbosch University, Stellenbosch, South Africa; 4 Department of Plant and Microbial Biology, University of California, Berkeley, Berkeley, California, United States of America; 5 Department of Infectious Disease Epidemiology, MRC Centre for Global Infectious Disease Analysis, White City, Imperial, London, United Kingdom; University of Melbourne, AUSTRALIA

## Introduction

**Fig 1:**
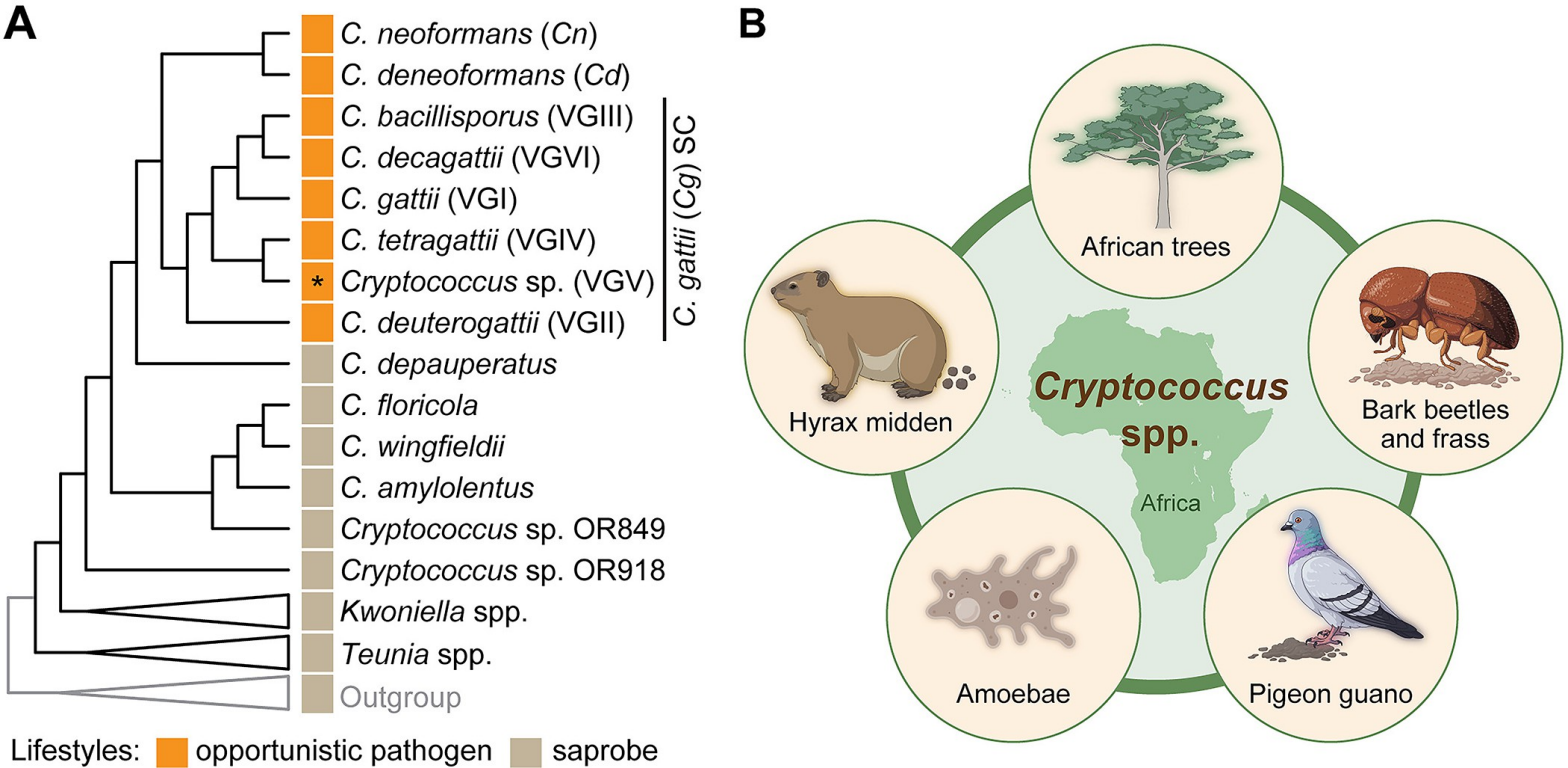
Phylogenetic relationships and possible environmental Interactions of *Cryptococcus* species in Africa. **(A)** Cladogram illustrating the phylogenetic relationships among *Cryptococcus* species and the related genera *Kwoniella* and *Teunia* within the family *Cryptococcaceae* (with branches for *Teunia* spp., *Kwoniella* spp., and the outgroup compressed for easier visualization; based on refs. [21,38]). Opportunistic pathogenic *Cryptococcus* species, including *C*. *neoformans* (*Cn*), *C*. *deneoformans* (*Cd*), and the *C*. *gattii* (*Cg*) species complex (SC) are highlighted in orange as indicated in the key. An asterisk (*) denotes that *Cg* VGV has not yet been implicated in human infection. **(B)** The diversity of *Cryptococcus* species on the African continent suggests that Africa is not only a hotspot of diversity but also a backdrop for *Cryptococcus* evolution, facilitated by interactions with various competitors (e.g., amoebae and soil bacteria), hosts, and dispersal vectors (plants, insects, birds, and mammals). Panel B was generated with BioRender (https://www.biorender.com/).

Fungal pathogens cause millions of infections and deaths annually, while also contributing to global food insecurity [1]. Among them, basidiomycete *Cryptococcus* species—particularly *C*. *neoformans* (*Cn*; previously *C*. *neoformans* var. *grubii*, serotype A; lineages VNI, VNII, VNBI, and VNBII), *C*. *deneoformans* (*Cd*; previously *C*. *neoformans* var. *neoformans*, serotype D; lineage VNIV), and the *C*. *gattii* (*Cg*) species complex (**Fig 1A**)—are significant opportunistic and primary pathogens, especially in sub-Saharan Africa [2,3]. These pathogens primarily cause cryptococcosis, manifesting as severe pulmonary infections or life-threatening meningoencephalitis in both immunocompromised and apparently immunocompetent individuals. Exposures are typically thought to occur by inhalation of desiccated yeast cells or spores from the environment [4]. While *Cryptococcus* species vary in their occurrence worldwide, mounting evidence suggests an evolutionary origin in Africa for most of the pathogenic *Cryptococcus* species, where they occupy diverse ecological niches such as trees, pigeon guano, and mammalian middens (**Fig 1B**). While *Cn*, *Cd*, and *Cg* are pathogenic, nonpathogenic species within the genus (such as *C*. *amylolentus*, *C*. *wingfieldii*, and *C*. *floricola*; **Fig 1A**) occur either as African microendemic species or are known thus far from only a single isolate in the Canary Islands (*C*. *floricola*) [5,6]. This review explores the likely African origins of *Cryptococcus*, its ecological diversity, and how pathogenic species spread globally, transitioning from environmental microbes to human pathogens.

## Where did pathogenic *Cryptococcus* species originate and where are they found today?

*Cg* and *Cn* occur globally, inhabiting all continents except Antarctica. The emergence of the virulent *Cg* VGIIa major genotype (*C*. *deuterogattii* VGII, subtype a) along the North American Pacific Northwest, triggering the Vancouver Island outbreak [7], spurred interest in tracing the origins of invasive *Cryptococcus* lineages alongside deeper reservoirs of species diversity [8,9]. Efforts to identify phylogeographic hotspots have focused on ancestral genotypes, high local-scale genetic diversity, the presence of both mating types, and patterns of recombination in natural populations.

Africa houses most of the global diversity for *Cn*, marked by the presence of the highly diverse VNBI and VNBII lineages, which include both mating types (**a** and α) alongside high rates of linkage disequilibrium decay [10], suggesting a long evolutionary history in the region. These lineages are highly prevalent on Mopane trees (*Colophospermum mopane*) in sub-Saharan Africa [11,12], with rare occurrences in South America [13] and on olive trees in Türkiye [14]. The *Cn* VNI lineage, however, is globally ubiquitous and highly pathogenic to humans, and the occurrence of nearly identical genotypes worldwide is evidence for widespread transcontinental dispersal [10], likely mediated by birds, particularly pigeons. Notably, *Cn* VNI exhibits a much stronger bias toward the α mating type compared to *Cn* VNBI and *Cn* VNBII, a feature likely associated with unisexual reproduction (mating between cells of the same mating type) [15], which may nonetheless occur across all the lineages.

In the *Cg* species complex, 6 phylogenetic species are recognized: *C*. *gattii* (VGI), *C*. *deuterogattii* (VGII), *C*. *bacillisporus* (VGIII), *C*. *tetragattii* (VGIV), *C*. *decagattii* (VGVI), and a recently discovered lineage provisionally named *C*. *gattii* VGV [16], which is the only *Cg* species that has not yet been linked to human infection (**Fig 1A**). These species have been recovered from African environments, with VGV (isolated in Zambia) being endemic to the continent, together pointing to a greater diversity of *Cg* in Africa than elsewhere. *Cryptococcus deuterogattii* (VGII), in particular, has been extensively studied owing to its role in the Vancouver Island outbreak. While multilocus sequence typing (MLST) analyses have not conclusively pinpointed its origin, South America, particularly Brazil, emerges as one possible source due to the high genetic diversity, recombination rates, and the presence of ancestral lineages in both the Amazon rainforest and semi-arid regions of Brazil [8,9]. Recent phylogenomic studies, however, have complicated this view by revealing ancestral VGII genotypes present at high frequency in both Africa (Zambia) and Australia [16]. Given the significant genetic diversity observed in South America and Africa, more extensive genomic sequencing of environmental isolates from both regions is needed to fully understand the evolutionary history of this species.

The balance of evidence, therefore, suggests Africa as the leading evolutionary cradle candidate for both *Cn* and most, if not all, *Cg* species, with diverse and ancestral lineages originating on the continent. Enhanced genomic surveillance of *Cryptococcus* in nature is needed to better understand the ecological and evolutionary processes shaping the current distributions of these pathogens worldwide. This environmental emphasis is highly relevant for Africa, where environmental sampling and sequencing have been sparse, despite the continent harboring the largest human population at risk from cryptococcosis owing to the ongoing high prevalence of immunocompromised people living with HIV/AIDS [3].

## Why are *Cn* lineages associated with pigeon guano and trees?

Association with birds and their guano is thought to have driven the global distribution of the *Cn* VNI global lineage. While the *Cn* VNBI and VNBII lineages are mainly found in Southern Africa, associated with Mopane trees, *Cn* VNI is closely associated with the guano of the globally distributed rock dove (*Columba livia*) [17], a bird species that itself evolved in North Africa and the Mediterranean basin [18]. Interestingly, the sister species *C*. *deneoformans* (serotype D, VNIV) is also frequently isolated from pigeon guano, raising the possibility that adaptation to this niche arose either independently in the 2 species through convergent evolution, possibly driven by the nutrient richness and selective pressures of this environment, or was introduced through introgression between the 2 species during their descent from the last common ancestor. Investigating substrate use among different *Cryptococcus* lineages and their relatives through comparative genomics could reveal how *Cn* VNI and *Cd* evolved to utilize pigeon guano by identifying adaptations for nutrient acquisition from this source.

*Cryptococcus* belongs to the class *Tremellomycetes*, family *Cryptococcaceae*, which also includes the genera *Kwoniella* and *Teunia* [19–21] (**Fig 1**). *Kwoniella* species are typically associated with trees, wood decay, and insects [22], while *Teunia* species have been isolated from various plant parts. Although the phylogenic backbone is not fully resolved, related lineages include *Phaeotremellaceae* fungi (e.g., *Tremella*) that parasitize fungal fruiting bodies [23,24], and *Trimorphomycetaceae* species (e.g., *Saitozyma*), which are soil saprobes that use decaying plant material as substrate [25]. The substrate preferences of these closely related non-*Cryptococcus* lineages, which tend to have narrower distributions than *Cn* and range from mycoparasitic to saprobic, suggest that *Cn* VNI achieved its global distributions when it evolved from deriving its nutrition through mycoparasitism or saprophytism of unaltered vegetation to basing it on pigeon guano [26,27].

*Cn* VNI has been isolated from both aged and freshly deposited pigeon guano [28–30], as well as from the beaks and cloacas of pigeons [30], but not from their internal organs [28,30]. Although pigeons can be lethally infected via intracerebral injection with high *Cn* inocula [31], there are no reports of systemic infections or deaths from *Cn* in wild pigeons. How might these results be reconciled? While high body temperature alone seems to be insufficient to prevent *Cn* growth, avian (chicken) macrophages at avian body temperatures (41 to 43°C) strongly suppress *Cn* proliferation [32]. This suppression suggests that inhaled spores and desiccated yeasts may be more effectively cleared by avian than mammalian alveolar macrophages. However, a small proportion of fungal cells can still escape killing (via vomocytosis), allowing pigeons to harbor low numbers of cryptococci for prolonged periods without developing disease [32]. As a result, pigeons likely act as long-range vectors, inoculating environments such as guano with the fungus while remaining largely uninfected. Although pigeons are the main avian associates, *Cn* has also been isolated from the cloacas and nests of raptor species, such as *Falco* and *Buteo* [33]. This observation, together with the finding that *Cn* VNI was not detected in a thorough survey of birds trapped in France, which did find nonpathogenic species closely related to *Cryptococcus* [34], raises the possibility that birds preying on pigeons might also help spread *Cn* VNI.

Knowing that wild pigeons feed on seeds, fruits, and other parts of plants and that urban pigeons augment this diet with refuse [35], *Cn* VNI may have adapted to derive nutrition from the gut contents of pigeons, rather than solely from plants or other fungi. While genomic comparisons between pathogenic and nonpathogenic *Cryptococcus* species have provided valuable insights into evolutionary adaptations (see later section, [37]), these studies have yet to fully explore the ecological differences across lineages and niches, as has been done with *Coccidioides* species [36,37]. Targeted comparative approaches, focusing on differences in functional categories associated with host or substrate shifts, such as metabolism and nutrient acquisition, could be especially informative. *Cn* grows robustly and reproduces sexually on pigeon guano media [26]. This provides a facile experimental system to explore how *Cn* VNI adapted to this niche, and why some of the other pathogenic lineages (*Cn* VNBI/VNBII and *Cg* species) and nonpathogenic species have not.

## How did *Cryptococcus* evolve to infect mammals?

Pathogenic *Cryptococcus* species evolved from a common ancestor shared with nonpathogenic environmental saprobes or mycoparasites, with pathogenicity emerging at least ~27 million years ago in the last common ancestor of the pathogenic clade [38]. Attributes important for virulence in mammals, such as capsule formation, melanin production, and urease activity, likely originally evolved because they enhanced survival of *Cryptococcus* in environmental predators including amoebae, nematodes, and insects [39,40]. Another key adaptation is the ability to survive and proliferate at mammalian body temperature (37°C). While the evolutionary pressures behind this thermal tolerance remain unclear, one possible selective force could have been survival during environmental temperature extremes. A similar evolutionary path was recently observed in *Rhodosporidiobolus fluvialis*, a red yeast that developed high-temperature tolerance and the ability to cause human infections [41].

The evolution of *Cryptococcus* pathogenicity in mammals may have involved circulation between infected mammals and the environment. Evolution may first have entailed survival on the skin, progressing to the nasopharynx or lungs before possible return to the environment through saliva or respiratory droplets. Mice infected with *Cryptococcus* have been reported to contaminate their bedding [42]. Thus, a host-environment-to-host cycle might have driven adaptive evolution, ultimately fixing virulence traits over time. Further comparisons of pathogenic *Cryptococcus* lineages that grow at 37°C with closely aligned nonpathogens that cannot grow above 32°C, coupled with experimental evolution studies for survival at increasing temperature, could help elucidate how *Cryptococcus* adapted to infect mammals.

Comparative genomics offers insights into the gene content differences between pathogenic and nonpathogenic *Cryptococcus* [38]. While many of the virulence-related genes are largely conserved across both groups [38], pathogenic species tend to have fewer genes overall, suggesting that gene loss may have contributed to (or been a consequence of) the evolution of pathogenic potential. A striking example is the loss of the zincophore Pra1 and its associated zinc transporter Zrt1 in pathogenic *Cryptococcus* species, while both of these are retained in nearly all nonpathogenic *Cryptococcus*/*Kwoniella* species [38]. In *Candida albicans*, Pra1 functions as a pathogen-associated molecular pattern (PAMP) recruiting neutrophils to infection sites [43]; thus, its loss in pathogenic *Cryptococcus* species may have helped evade immune detection, enhancing survival during early stages of lung colonization. Previous comparative genomics studies of *Coccidioides immitis* with closely aligned nonpathogenic species revealed a dramatic loss of genes for carbohydrate utilization and a concomitant expansion of protein degradation genes, reflecting a shift from a plant-centric to an animal-centric lifestyle [36]. That a similar pattern was not observed in *Cryptococcus* [38] suggests that it followed an alternative evolutionary trajectory from environmental saprobes to successful human fungal pathogens.

Studies in heterologous hosts such as *Drosophila*, *Galleria*, and *Caenorhabditis elegans* provide further insights into *Cryptococcus* pathogenesis, as traits that promote pathogenesis in mice and other mammals often also enhance survival in these models [44]. However, some limitations lie in not fully replicating the mammalian immune environment, especially regarding thermal tolerance. Survival in amoebae, which are naturally phagocytic like macrophages, has been proposed as a staging ground for the evolution of fungal pathogenesis in mammals [45]. Yet, *Cryptococcus* evolution for enhanced survival in amoebae is not correlated with virulence in mammals [40], and can even reduce virulence due to impaired growth at high temperature, as seen in RAM pathway mutants [46]. One limitation of these studies is that *Cryptococcus* survival in amoebae was selected or assessed at lower growth temperatures (25 and 30°C). Future studies could focus on selecting for survival in amoeba at 37°C, or in amoeba species with higher temperature tolerance, and then test for impact on mammalian pathogenesis. A recent experimental evolution study showed that a partial loss-of-function mutation in adenylyl cyclase enhanced survival in macrophages but reduced pathogenesis in mice, again illustrating distinct evolutionary trajectories that do not translate into increased pathogenesis in mammals [47].

## Could *Cryptococcus* pathogen lineages be associated with African tree-infesting bark beetles?

Various *Cryptococcus* species have been associated with insects and their frass [48–50], including tree-infesting bark beetles (*Coleoptera*: *Scolytidae*) [51,52]. An intriguing example is *C*. *wingfieldii*, a yeast first isolated in 1987 from an unidentified twig-feeding bark beetle on the African olive tree (*Olea europaea*). Initially named *Sterigmatomyces wingfieldii* [53], it was later reclassified as *Tsuchiyaea wingfieldii* based on chemotaxonomy (Q-9) and morphological characteristics [54]. More recent DNA-based studies, including detailed genetic and genomic analysis, placed this species within *Cryptococcus*, showing a close relationship to *C*. *amylolentus* (from beetle frass in South Africa) and *C*. *floricola* (from flower nectar in Tenerife, Canary Islands) [5,20,38,55] (**Fig 1A**). Together, these species form a nonpathogenic lineage closely related to the lineage of human pathogenic *Cryptococcus* [5,20,38,55]. This discovery raised, for the first time, the possibility that *Cryptococcus* species could have a close and previously unrecognized ecological association with tree-infesting bark beetles in Africa.

Recent work by Basson and colleagues [56] seems to support this hypothesis. Fungi were isolated from bark beetles, representing undescribed species of *Lanurgus* [57], the mainly South African genus whose species infect the iconic and threatened conifer, *Widdringtonia cedarbergensis* (= *W*. *wallichii*), in the Cederberg mountains of South Africa. *Cryptococcus* isolates were frequently obtained from two of these beetle species, both from the insects themselves and their frass. DNA sequence data places these isolates within the *C*. *wingfieldii/C*. *amylolentus/C*. *floricola* clade [56]. Together with the earlier finding of *C*. *wingfieldii* from a beetle infesting Cape wild olive [53], the collections from beetles/frass on *W*. *cedarbergensis* provides further clues of a potential ecological relationship between *Cryptococcus* and tree-infesting bark beetles.

Bark beetle frass has a powdery consistency that is wind-borne and serves as an important inoculum source for dispersal of various insect-associated fungi [58,59]. This feature suggests that *Cryptococcus* species found in the beetle frass could be widely distributed in wind currents, potentially reaching the surfaces of any number of substrates in the environment, including trees, soil, and bird guano. Given the established connections of *Cn* VNBI/II lineages with Southern Africa Mopane trees [17], as well as the associations of *Cg* lineages with various trees [60–62], further research should explore whether insects, particularly tree-infesting bark beetles, play a role in mediating these fungal-arboreal interactions. Understanding these ecological dynamics could provide valuable insights into how pathogenic *Cryptococcus* lineages are dispersed and maintained in nature.

## Implications and outlook

Pathogenic *Cryptococcus* species have complex ecological and evolutionary histories that trace back to Africa, with strong associations with trees, birds, and possibly insects (**Fig 1B**). Their evolution from environmental saprobes or mycoparasites to human pathogens involved key adaptations, including thermal tolerance and likely gene loss. Understanding the ecological roles of *Cryptococcus* species in natural settings, especially in relation to pigeon guano and insect vectors like bark beetles, will be critical in unraveling the dynamics of their global spread and pathogenicity. Enhanced genomic surveillance and experimental studies will continue to illuminate these evolutionary trajectories, ultimately aiding in the development of strategies to mitigate the impact of cryptococcosis, particularly in vulnerable populations across Africa.
